# Target-Specific Oral Anticoagulants—New Approaches in the Field of Oral Anticoagulation

**DOI:** 10.1155/2015/549803

**Published:** 2015-05-20

**Authors:** Helen Mani, Jonathan Douxfils, Jack Ansell

**Affiliations:** ^1^Hemostasis Center, Department of Internal Medicine, University Hospital Frankfurt, 60590 Frankfurt, Germany; ^2^Department of Pharmacy, Namur Research Institute for Life Sciences (NARLIS), University of Namur, 5000 Namur, Belgium; ^3^Hofstra North Shore-LIJ School of Medicine, Hofstra Boulevard, Hempstead, NY 11549, USA

Direct oral anticoagulants targeting specific coagulation factors have been introduced as alternatives to conventional anticoagulants for both prophylactic and therapeutic indications. They have succeeded in overcoming the limitations of vitamin K antagonists.

Although the favourable efficacy and side-effect profiles compared to vitamin K antagonists is proven, there is significant concern about management of hemorrhage under direct oral anticoagulants. There is no specific antidote or method for reversing the antithrombotic effect of these drugs available, at present. Most of the management of hemorrhage is based on expert opinion and case reviews. Specific treatment regimens are needed to avoid consequences of acute hemorrhage while patients are on anticoagulation. Therefore, the focus of this special issue is to define a management system for oral anticoagulants in the peri- and postoperative setting.

Moreover, the long-term safety in clinical routine life of direct oral anticoagulants is not well-known. One study performed in clinical practice included a small cohort of patients with acute cerebral hemorrhage starting dabigatran etexilate for secondary stroke prevention to investigate efficacy and safety of this drug for one year. Another point which has to be considered for treatment with direct oral anticoagulants depends on the cost-effectiveness. In this issue a comparison of the cost-utility analysis between edoxaban, dabigatran, rivaroxaban, and apixaban in German population is described.

Due to the heterogeneous mode of action and pharmacokinetic profile, each direct oral anticoagulant will vary in its effects on laboratory assays, and to avoid mismanagement clinicians are placed in a difficult position. One review published in this issue provides an overview of current knowledge regarding appropriate coagulation assays to measure the pharmacodynamics of the direct oral anticoagulants.

For this special issue we have also invited investigators to contribute original research articles that continue the development of strategies performed by the direct oral anticoagulants. The synthesis of a novel compound, trehalose octasulfate, is presented that employs a multitarget strategy to modulate the activity of various components of thrombus formation.

Good quality observational studies and data analyses are often sufficient to provide a guide for better treatment with drugs as the new anticoagulants. Different models of anticoagulation treatment might have different impacts on patients' satisfaction. However, more investigation is needed on the management of the target-specific oral anticoagulants.

